# 1239. Ceftaroline versus Vancomycin as First-Line Therapy for MRSA Bacteremia

**DOI:** 10.1093/ofid/ofab466.1431

**Published:** 2021-12-04

**Authors:** Meghan Kamath, Ariel Ma, Scott T Johns

**Affiliations:** 1 VA San Diego Healthcare System, San Diego, California; 2 VA San Diego Medical Center, San Diego, California; 3 San Diego VA Healthcare System, San Diego, California

## Abstract

**Background:**

Beta-lactams have demonstrated superior outcomes over vancomycin in MSSA bacteremia. Despite this, studies of the anti-MRSA beta-lactam ceftaroline in MRSA bacteremia (MRSAB) are largely limited in size or focus on combination or salvage regimens. This study sought to further examine ceftaroline as first-line therapy for MRSAB.

**Methods:**

This was a retrospective matched cohort study at the San Diego VA Medical Center between November 2010 and June 2020. Patients had to have received at least 72 hours of ceftaroline or vancomycin for MRSAB and less than 72 hours of prior MRSA therapy. Adjunct MRSA therapy was allowed only if routinely indicated for the infection (e.g. rifampin for prosthesis). Patients in the vancomycin group were matched 1:1 to patients in the ceftaroline group by age (+/- 10 years) and Pitt bacteremia score (+/- 1 point). The primary outcome was duration of bacteremia after initiation of MRSA therapy, including time on prior MRSA therapy.

**Results:**

Fifteen patients were included in each group, with a median age of 65 years and Pitt bacteremia score of 0. Patients in the ceftaroline group were more likely to have CKD; to have been on a different MRSA agent prior to initiation of the study drug, with a median of 1 day of prior treatment; and to have been on adjunctive rifampin or clindamycin. Though not significant, more patients in the ceftaroline group also had endovascular sources, uncontrolled sources, and longer durations of therapy. The median duration of bacteremia after initiation of MRSA therapy did not significantly differ between ceftaroline and vancomycin (4 vs. 3 days, p = 0.806). In addition, 30-day all-cause mortality, in-hospital mortality, 90-day readmission or treatment failure, inpatient length of stay, total duration of bacteremia, and rate of adverse events did not significantly differ between groups.

**Conclusion:**

This study suggests ceftaroline may be an appropriate first-line agent for the treatment of MRSA bacteremia with similar outcomes between groups despite the ceftaroline group likely experiencing more difficult-to-treat infections. However, it was not powered to detect differences between groups, and its retrospective nature has the potential to introduce bias. Prospective comparative studies are needed to corroborate these findings.

**Disclosures:**

**All Authors**: No reported disclosures

